# Precursor of Advanced Glycation End Products Mediates ER-Stress-Induced Caspase-3 Activation of Human Dermal Fibroblasts through NAD(P)H Oxidase 4

**DOI:** 10.1371/journal.pone.0011093

**Published:** 2010-06-14

**Authors:** Danielle T. Loughlin, Carol M. Artlett

**Affiliations:** Microbiology and Immunology, Drexel University College of Medicine, Philadelphia, Pennsylvania, United States of America; University of Dayton, United States of America

## Abstract

**Background:**

The precursor for advanced glycation end products, 3-deoxyglucosone (3DG) is highly upregulated in skin explants of diabetic cutaneous wounds, and has been shown to negatively impact dermal fibroblasts, which are crucial in wound remodeling. 3DG induces apoptosis however; the mechanisms involved in the apoptotic action of 3DG in the pathogenesis of diabetic chronic wounds are poorly understood. Therefore, we sought to delineate novel mechanisms involved with the 3DG-collagen induced apoptosis.

**Methodology/Principal Findings:**

Using human dermal fibroblasts, we demonstrated that 3DG-modified collagen induces oxidative stress and caspase-3 activation. Oxidative stress was found to be dependent on the upregulation of NAD(P)H oxidase 4 (Nox4), a reactive oxygen species (ROS) Nox homologue, triggering endoplasmic reticulum (ER) stress, as assessed by the ER stress-induced apoptosis marker Growth Arrest and DNA Damage-inducible gene 153 (GADD153). We demonstrated that 3DG-collagen activated GADD153 via phosphorylation of p38 mitogen activated protein kinase (MAPK), and this was dependent on upstream ROS. Inhibition of ROS and/or p38 MAPK abrogated 3DG-collagen induced caspase-3 activation. Our investigations also demonstrated that 3DG-collagen-induced caspase-3 activation did not signal through the canonical receptor for advanced glycation end products (RAGE) but through integrin α1β1. To further verify the role of integrins, neutralization of integrins α1β1 prevented 3DG-collagen-induced upregulation of ROS, GADD153, and caspase-3 activation; suggesting that 3DG-collagen signaling to the fibroblast is dependent on integrins α1β1.

**Conclusions/Significance:**

Taken together, these findings demonstrate for the first time that a RAGE independent mechanism is involved in 3DG-collagen-induced apoptosis. Moreover, the ER stress pathway through activation of Nox4 by integrins α1β1 plays a key role in 3DG-collagen-induced caspase-3 activation, which may play an important role in the pathogenesis of diabetic wounds.

## Introduction

Wound healing is impaired in patients with diabetes as approximately 5–8% of the patients develop chronic foot ulcers [1]–[3]. Patients suffering from chronic diabetic ulcers have increased levels of apoptosis within their infected tissues and this may interfere with their capacity to efficiently heal wounds [1], [3]–[7]. The high degree of cell death seen in fibroblasts explanted from the skin of diabetic patients may be partly due to the formation of advanced glycation end products (AGEs) [8]. AGEs result from a non-enzymatic reaction between glucose and protein, which can form irreversible cross-links on long-lived proteins such as collagen [6], [9]–[11]. One precursor for AGEs is the highly reactive α-dicarbonyl 3-deoxyglucosone (3DG). 3DG has been shown to play a role in the modification and cross-linking of collagen [9], [10], [12], [13]. One mechanism by which 3DG-modifed collagen could affect wound healing is through increased apoptosis.

The role of AGE induction of apoptosis has been investigated extensively, however many of these studies were performed on various cell types including neuronal and endothelial cells [14]–[20] using soluble AGEs, resulting in differing apoptotic responses [8], [20]–[22]. Soluble AGEs induce apoptosis in endothelial and neuronal cells through engagement of its receptor, receptor for advanced glycation end products (RAGE) [15]–[20]. This ligand-receptor engagement promotes the upregulation of reactive oxygen species (ROS) resulting in apoptosis [19], [23]–[25]. Moreover, reports utilizing AGEs cross-linked to collagen agree with the current view that AGEs induce apoptosis; however, the mechanisms by which AGE-modifed collagen does so are conflicting and not well understood [8]. More importantly, none of the studies to date have investigated 3DG or its signaling events that induce apoptosis in dermal fibroblasts.

One possibility for the conflicting evidence is due to the varying types of precursors responsible for the development of AGEs [9], [10], [26]. We previously demonstrated that 3DG-collagen signals to the fibroblast in an anti-fibrotic way, causing decreased fibroblast migration, proliferation, and extracellular matrix (ECM) production [27]–[29]. In contrast, methylglyoxal (MG), a well studied AGE precursor, has been implicated in pro-fibrotic conditions such as atherosclerosis, and modification of collagen by MG has been shown to increase fibroblast proliferation and ECM production [10], [29]–[31]. Because the varying pathology of the AGEs in diabetes could rely on the dicarbonyl that produces them, it is important to understand the role of apoptosis in the context of independent AGE precursors. Therefore, we investigated the mechanism through which 3DG-collagen induces apoptosis in human dermal fibroblasts.

Previous work in our laboratory has revealed a role for the endoplasmic reticulum (ER) stress signaling pathway in promoting apoptosis of dermal fibroblasts cultured on 3DG-collagen [28]. ER stress can be induced by various stressors on the cell including oxidative stress [32]–[37]. ROS can be formed through activation of the NAD(P)H oxidase system [38]–[40] and imbalanced activation of this oxidase system can lead to oxidative stress [39], [41]. ROS have been shown to disrupt ER homeostasis resulting in the accumulation of misfolded proteins and induction of ER stress [35]–[37], [42]. ROS induces the transcription factor Growth Arrest and DNA Damage inducible gene 153 (GADD153), also known as C/EBP homologous protein, through phosphorylation of the stress activated kinase p38 MAPK [35], [37], [43]–[45], where its activation is considered to be a classic marker of ER stress-induced apoptosis [33], [34], [43], [46]–[48]. Induction of GADD153 causes the cell to undergo apoptosis through the upregulation of caspase-3 [37], [43]. Upon investigation, GADD153 was found to be highly upregulated in fibroblasts cultured on 3DG-collagen, while its expression was downregulated in fibroblasts treated with the 3DG inhibitor meglumine [28]. To date, there are no reports suggesting a possible mechanism by which 3DG-collagen induces the apoptosis signaling cascade in dermal fibroblasts. Therefore, this study was undertaken to further delineate the 3DG-collagen signaling mechanism with emphasis on the ER stress pathway.

## Results

### ER stress mediates 3DG-collagen-induced caspase-3 activation in human dermal fibroblasts

**Figure 1:**
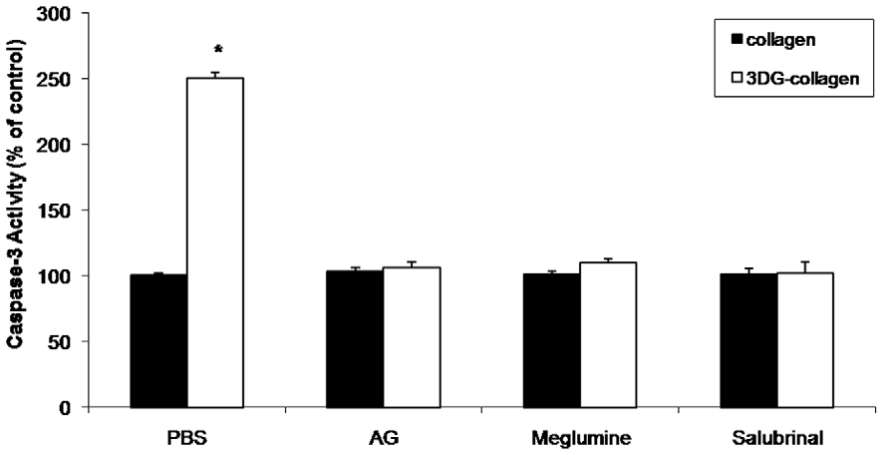
Induction of caspase-3 activity by 3DG-collagen is dependent on ER stress. Fibroblasts were cultured on native collagen or 3DG-collagen and treated with or without 5 mM AG, 40 mM meglumine, or 40 µM salubrinal for 24 h. 100 µg of whole cell lysate was assayed for caspase-3 activity according to the protocol from Caspase-3 Colorimetric Correlate Assay. All samples were performed in triplicate and normalized to the control samples. All comparisons are made against collagen treated with PBS. Data are mean±SD (n = 3), *P<0.0002.

To test whether 3DG-collagen induces the apoptotic signaling cascade in human dermal fibroblasts and determine if it was dependent on ER stress, fibroblasts were cultured on native collagen or 3DG-collagen coated dishes with or without aminoguanidine (AG), or treated with or without meglumine for 24 h. Apoptosis was measured by the expression of active caspase-3, an early marker of apoptosis. Fibroblasts cultured on 3DG-collagen induced a 150%±4.5% increase in active caspase-3 expression compared to fibroblasts grown on native collagen (Figure 1, p<0.0002). When fibroblasts were treated with AG, which is known to inactivate 3DG's ability to cross-link collagen, the activity of caspase-3 was reduced to levels observed in fibroblasts grown on native collagen. Additionally, meglumine, an inhibitor of 3DG, prevented the 3DG-collagen-induced increase in caspase-3 activity (Figure 1).

ER stress is known to induce apoptosis of the cell [32]–[35], [37], [43], [46], [48]. Therefore, fibroblasts were pretreated with the ER stress inhibitor salubrinal and then cultured on native collagen or 3DG-collagen for 24 h. Treatment with salubrinal reduced the level of active caspase-3 within fibroblasts cultured on 3DG-collagen to that of fibroblasts cultured on native collagen (Figure 1, p<0.0002). These results suggest that 3DG-collagen can induce the activation of the apoptotic signaling cascade through ER stress.

### ER stress-induced apoptosis marker GADD153 is upregulated in fibroblasts cultured on 3DG-collagen

**Figure 2:**
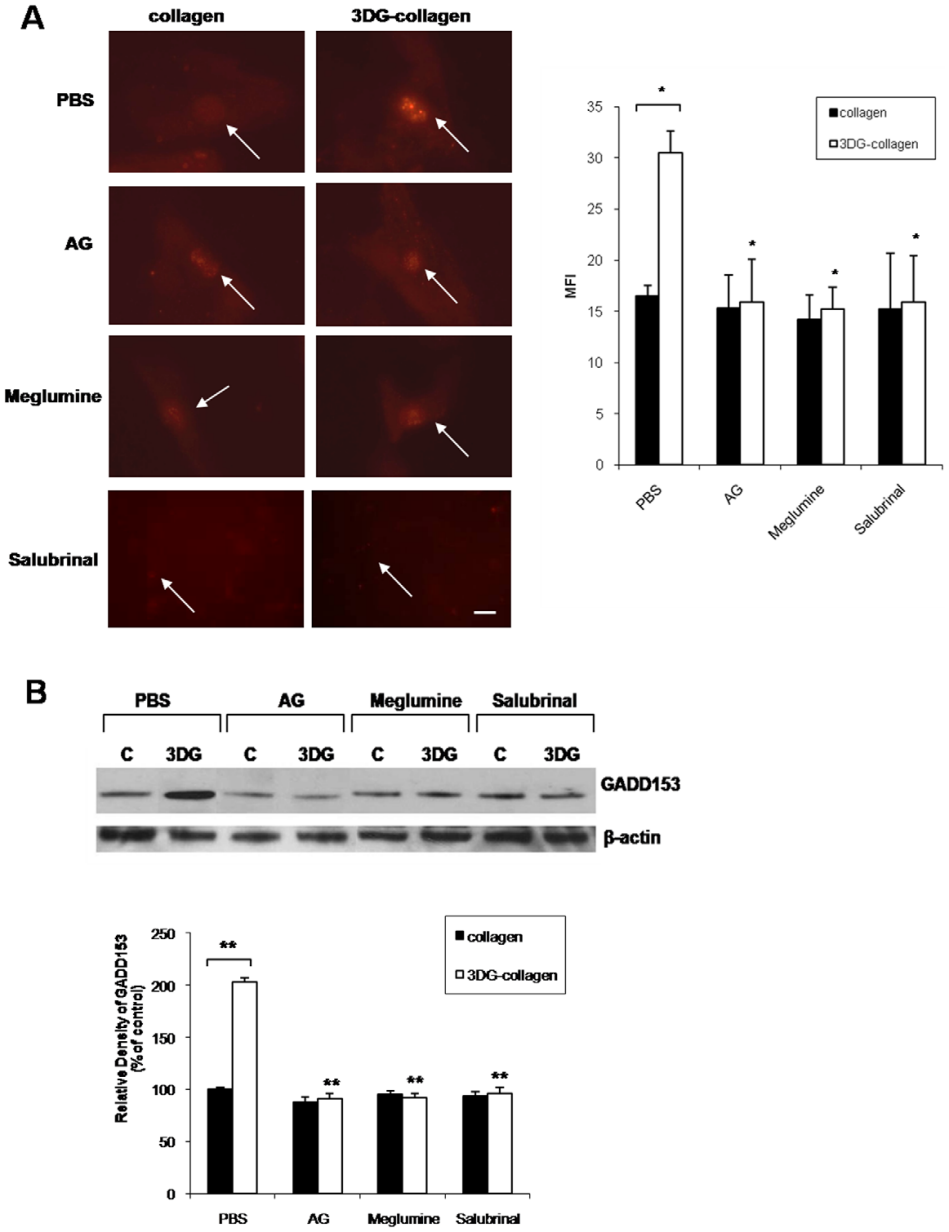
Effect of ER stress inhibitor salubrinal on 3DG-collagen-induced GADD153 expression. **A**, Fibroblasts were cultured in chamber slides coated with native collagen or 1 mM 3DG-collagen with or without 5 mM AG or 40 mM meglumine for 24 h. Also, fibroblasts were pretreated for 1 h with or without 40 µM salubrinal and then cultured on native collagen or 3DG-collagen for 24 h. Fibroblasts were stained and analyzed for expression of GADD153 in the nucleus by immunofluorescence analysis using Cy3-conjugated secondary antibody. Mean fluorescence intensity (MFI) of GADD153 in the nucleus was measured using ImageJ from ten representative fibroblasts. Images were taken at 40× magnification on an epi-fluorescent microscope. Arrows indicate nuclei containing GADD153. The bars represent the MFI values from each experimental condition. Scale bar represents 10 µm. **B**, Fibroblasts were treated as in **A** followed by Western blot for GADD153 expression. β-actin was used as a loading control. The bars represent the densitometric value for each experimental condition. All comparisons are made against 3DG-collagen treated with PBS unless otherwise indicated. Data are mean±SD (n = 3), **P<0.0005, *P<0.007.

Accumulation of misfolded proteins within the ER can lead to stress and induction of GADD153, a transcription factor involved in apoptosis [32]–[35], [37], [43], [46]–[48]. Previously, we demonstrated the upregulation of GADD153 transcript levels and activation of GADD153 in fibroblasts by 3DG-collagen [28]. Moreover, meglumine was found to inhibit 3DG-collagen-induced GADD153 expression [28]. To further confirm that 3DG-collagen is inducing ER stress, fibroblasts were pretreated with salubrinal, an inhibitor of ER stress, and cultured on 3DG-collagen for 24 h. The fibroblasts were then stained for GADD153 and inspected for GADD153 localization within the nucleus, which is indicative of activated GADD153 [27] and mean fluorescent intensity (MFI) of the nuclei was measured. In the presence of salubrinal, fibroblasts cultured on 3DG-collagen reduced the expression of GADD153 to that observed in fibroblasts cultured on native collagen (11.2 MFI±2.0 with salubrinal compared to 30.5 MFI±2.1 on 3DG-collagen), further confirming that 3DG-collagen is inducing ER stress in dermal fibroblasts (Figure 2A, p<0.007). To verify the immunofluorescence results, a Western blot was performed. Confirming the immunofluorescence observations, Western blot analysis demonstrated an increase to 203%±4.1% in GADD153 expression in fibroblasts cultured on 3DG-collagen compared to fibroblasts cultured on native collagen (p<0.0005). AG and meglumine reduced the level of GADD153 expression in fibroblasts cultured on 3DG-collagen to 91%±5.2% and 92%±4.2% (p<0.0005), respectively and salubrinal prevented the 3DG-collagen-induced increase of GADD153 (p<0.0005) (Figure 2B). These findings suggest that 3DG-collagen is inducing apoptosis through the ER stress-signaling pathway, which is dependent on GADD153 activation.

### 3DG-collagen stimulates ROS in dermal fibroblasts

**Figure 3:**
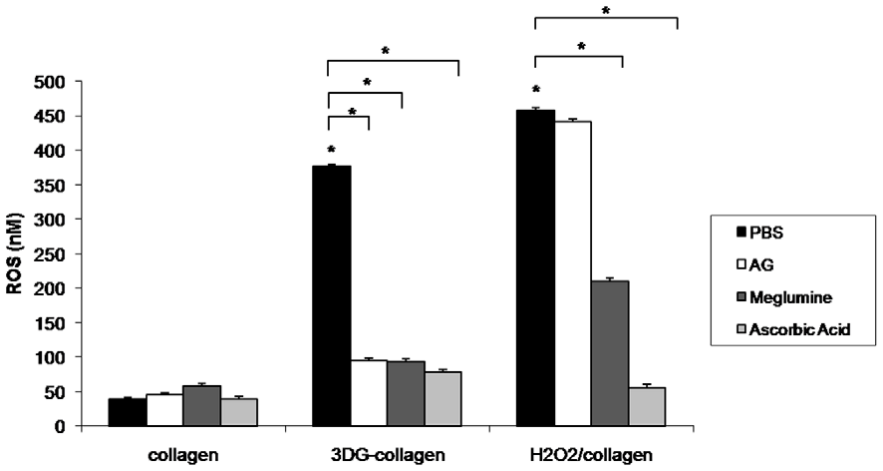
3DG-collagen stimulates intracellular ROS in fibroblasts. Fibroblasts were cultured in 96-well plate coated with either native collagen or 1 mM 3DG-collagen or treated with or without 5 mM AG, 40 mM meglumine, or 100 µg/mL ascorbic acid for 24 h. Treatment of fibroblasts cultured on native collagen with 50 µM H_2_O_2_ was used as a positive control. Fibroblasts were loaded with DCFH-DA for 30 min and ROS production was measured by absorbance of fluorescent DCF at a wavelength of 480 nm/530 nm. Comparisons are made to collagen treated with PBS unless otherwise indicated. Data are mean±SD (n = 3), *P<0.001.

ROS are known to cause oxidative stress and have been linked to the activation of GADD153-induced apoptosis in cells [35], [41], [43], [46], [49], [50]. Therefore, we determined if ROS were produced during the culturing of fibroblasts on 3DG-collagen. Fibroblasts cultured on 3DG-collagen produced 376 nM±3.4 of intracellular ROS at 24 h in comparison to the 38.7 nM±2.2 of ROS produced by fibroblasts grown on native collagen (Figure 3, p<0.001). This increase was comparable to that observed with hydrogen peroxide (H_2_O_2_), a free radical involved in ER stress, which produced 458 nM±3.2 (Figure 3, p<0.001). Moreover, AG abrogated the rise in ROS only in fibroblasts cultured on 3DG-collagen, and not in cells treated with H_2_O_2_ suggesting that 3DG-collagen is specifically producing ROS (Figure 3; 94.3 nM±4.0 for 3DG-collagen/AG; 440.6 nM±4.0 for H_2_O_2_/AG, p<0.001). Meglumine inhibited the production of ROS in cells cultured on 3DG-collagen and partially inhibited ROS in cells treated with H_2_O_2_ suggesting that meglumine may prevent ROS induction by 3DG-collagen and/or may be a scavenger of free radicals (92.8 nM±4.0 for 3DG-collagen and meglumine; and 210.2 nM±4.4 for H_2_O_2_ and meglumine, p<0.001). In addition, the induction of ROS by fibroblasts cultured on 3DG-collagen, or treated with H_2_O_2_ could be blocked by pretreating fibroblasts with the antioxidant ascorbic acid (Figure 3; 78.2 nM±3.6 for 3DG-collagen/ascorbic acid; and 55.6 nM±4.8 for H_2_O_2_/ascorbic acid, p<0.001). Taken together, these results suggest that ROS could be produced in the fibroblast in response to the modification of collagen by 3DG.

### NAD(P)H oxidase 4 is responsible for the 3DG-collagen-dependent production of ROS

**Figure 4:**
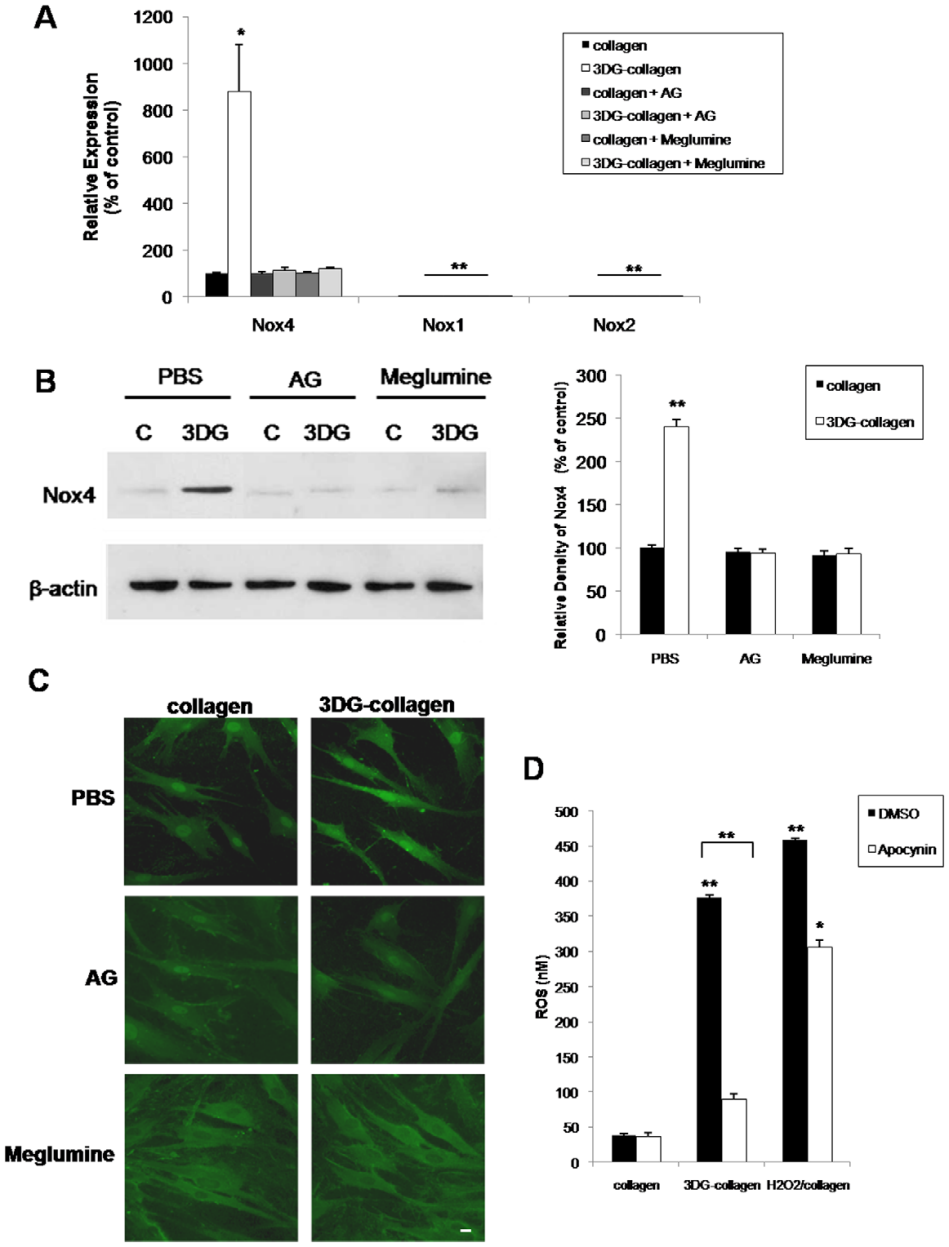
3DG-collagen increases expression of Nox4 in the dermal fibroblast. Fibroblasts were cultured on either native collagen or 1 mM 3DG-collagen and treated with or without 5 mM AG or 40 mM meglumine for 24 h. **A**, Nox4, Nox1, and Nox2 mRNA expression levels were quantified by real-time PCR. All transcripts were normalized to β-actin. **B**, Expression levels of Nox4 were analyzed by Western blot and β-actin served as a loading control. Results were quantified by densitometric scanning of the Western blot and normalized for β-actin. **C**, Localization of Nox4 in fibroblasts treated the same as in **A** and **B** was analyzed by immunofluorescence with the anti-Nox4 polyclonal antibody and Cy2-conjugated secondary antibody. Images were taken at 40× magnification on an epi-fluorescence microscope. Scale bar represents 10 µm. **D**, Inhibition of Nox4 reduces the level of intracellular ROS. Fibroblasts were pretreated for 1 h with either vehicle, DMSO or NOX inhibitor, apocynin (1 mM) and cultured on native collagen, 1 mM 3DG-collagen, or cultured on native collagen and treated with 50 µM H_2_O_2_ for 24 h. Fibroblasts were then incubated with DCFH-DA for 30 min and the level of intracellular ROS was determined by measuring the fluorescence at 480 nm/530 nm. Comparisons are made against collagen treated with DMSO and/or PBS unless otherwise indicated. Data are mean±SD (n = 3), **P<0.001, *P<0.02.

The NAD(P)H oxidase (Nox) controls the production of ROS through integrin activation, and cytokine and growth factor stimulation [38], [40], [51], [52]. Overexpression of key oxidases such as the non-phagocytic Nox4 has been associated with increased ROS and apoptosis [38], [39], [53]. Nox4 has been shown to be highly expressed in fibroblasts compared to other Nox homologues [38], [39]. Therefore, we determined if 3DG-collagen-induced ROS were mediated by the overexpression of Nox4. Quantitative real-time PCR revealed that Nox4 mRNA expression increased to 880%±200.0% in fibroblasts cultured on 3DG-collagen for 24 h compared to fibroblasts cultured on native collagen (Figure 4A, p<0.02). Moreover, to ensure that Nox4 was the only Nox isoform being overexpressed by 3DG-collagen, quantitative real-time PCR was performed to determine the mRNA transcript levels of the other Nox isoforms, Nox1 and Nox2. Detection of Nox1 and Nox2 mRNA transcripts was not apparent suggesting that dermal fibroblasts overexpress specifically Nox4 (Figure 4A). To show specificity of 3DG, AG and meglumine reduced the transcript levels of Nox4 in fibroblasts cultured on 3DG-collagen to that observed in fibroblasts cultured on native collagen. Additionally, Nox4 protein levels were found to be increased in fibroblasts cultured on 3DG-collagen compared to fibroblasts cultured on native collagen (240%±8.6% in 3DG-collagen treated vs. 100%±3.3% in native collagen treated cells, Figure 4B, p<0.001). This upregulation was also abrogated by the 3DG inhibitors AG and meglumine. Immunofluorescence demonstrated increased Nox4 localization at the plasma membrane in fibroblasts cultured on 3DG-collagen and that this increase was abrogated by AG and meglumine, suggesting that Nox4 may be activated on the cell surface (Figure 4C).

We further investigated the role of Nox4 in the upregulation of ROS in fibroblasts cultured on 3DG-collagen. Fibroblasts were pretreated with apocynin, a broad class Nox inhibitor, cultured on either native collagen, 3DG-collagen, or treated with H_2_O_2_ for 24 h, and intracellular ROS was quantified. Apocynin reduced ROS in fibroblasts cultured on 3DG-collagen to that observed in fibroblasts cultured on native collagen (Figure 4D, p<0.001). This further confirms that 3DG-collagen is inducing ROS through activation of Nox4. Furthermore, apocynin was found to only partially inhibit the level of ROS in fibroblasts cultured on native collagen with H_2_O_2_ suggesting that apocynin is inhibiting the ROS induced by the Nox4 complex rather than affecting the induction of ROS by exogenous H_2_O_2_ (Figure 4D, p<0.02).

### 3DG-collagen-induced phosphorylation of p38 MAPK is dependent on upstream ROS

**Figure 5:**
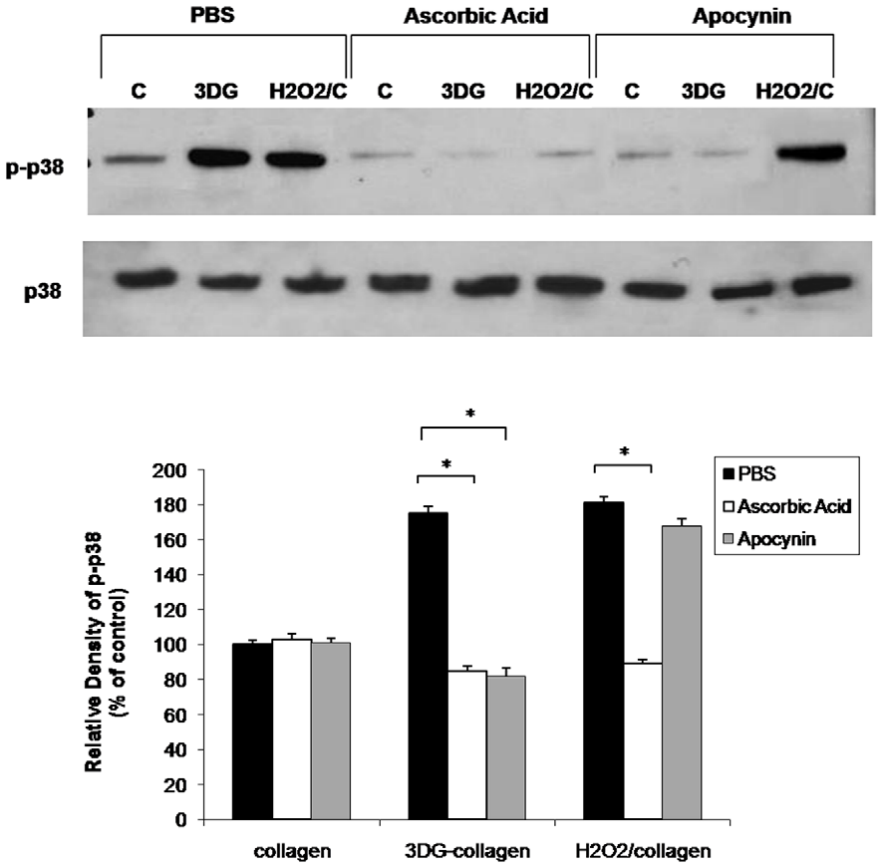
Phosphorylation of p38 MAPK is dependent on 3DG-collagen-induced ROS. Fibroblasts were pretreated for 1 h with 100 µg/mL of ascorbic acid, 1 mM apocynin, or DMSO and cultured on either native collagen, 1 mM 3DG-collagen, or treated with 50 µM H_2_O_2_ for 24 h. Whole cell lysates were extracted and Western blot analysis of p-p38 MAPK was performed. Total p38 MAPK was used as a loading control. The bars correspond to the densitometric value of p-p38 MAP kinase after normalization for total p38 MAP kinase. Data are mean±SD (n = 3), *P<0.0001.

During times of ER stress, ROS have been shown to activate the stress kinase p38 MAPK [35], [37], [44]. To determine if 3DG-collagen-induced ROS are responsible for increased phosphorylation of p38 MAPK, fibroblasts were pretreated with the antioxidant ascorbic acid and the Nox inhibitor apocynin, and cultured on either native collagen, 3DG-collagen, or native collagen and treated with H_2_O_2_ and protein levels were measured by Western blotting. Fibroblasts cultured on 3DG-collagen increased the phosphorylation of p38 MAPK to 175%±4.1%. As a positive control for ROS-induced p38 MAPK activation, fibroblasts cultured on native collagen and treated with H_2_O_2_ increased the phosphorylated p38 MAPK to 181%±3.3%. Pretreatment with ascorbic acid reduced the phosphorylation of p38 MAPK in fibroblasts cultured on 3DG-collagen or treated with H_2_O_2_ to that seen in fibroblasts cultured on native collagen (Figure 5, p<0.0001). Additionally pretreatment with apocynin reduced the level of phosphorylated p38 MAPK in fibroblasts cultured on 3DG-collagen, but not in fibroblasts treated with H_2_O_2_ (Figure 5, p<0.0001). These results suggest that 3DG-collagen-induced p38 MAPK is dependent on upstream production of ROS by Nox4.

### 3DG-collagen-induced GADD153 expression is dependent on upstream ROS and p38 MAPK activation

**Figure 6:**
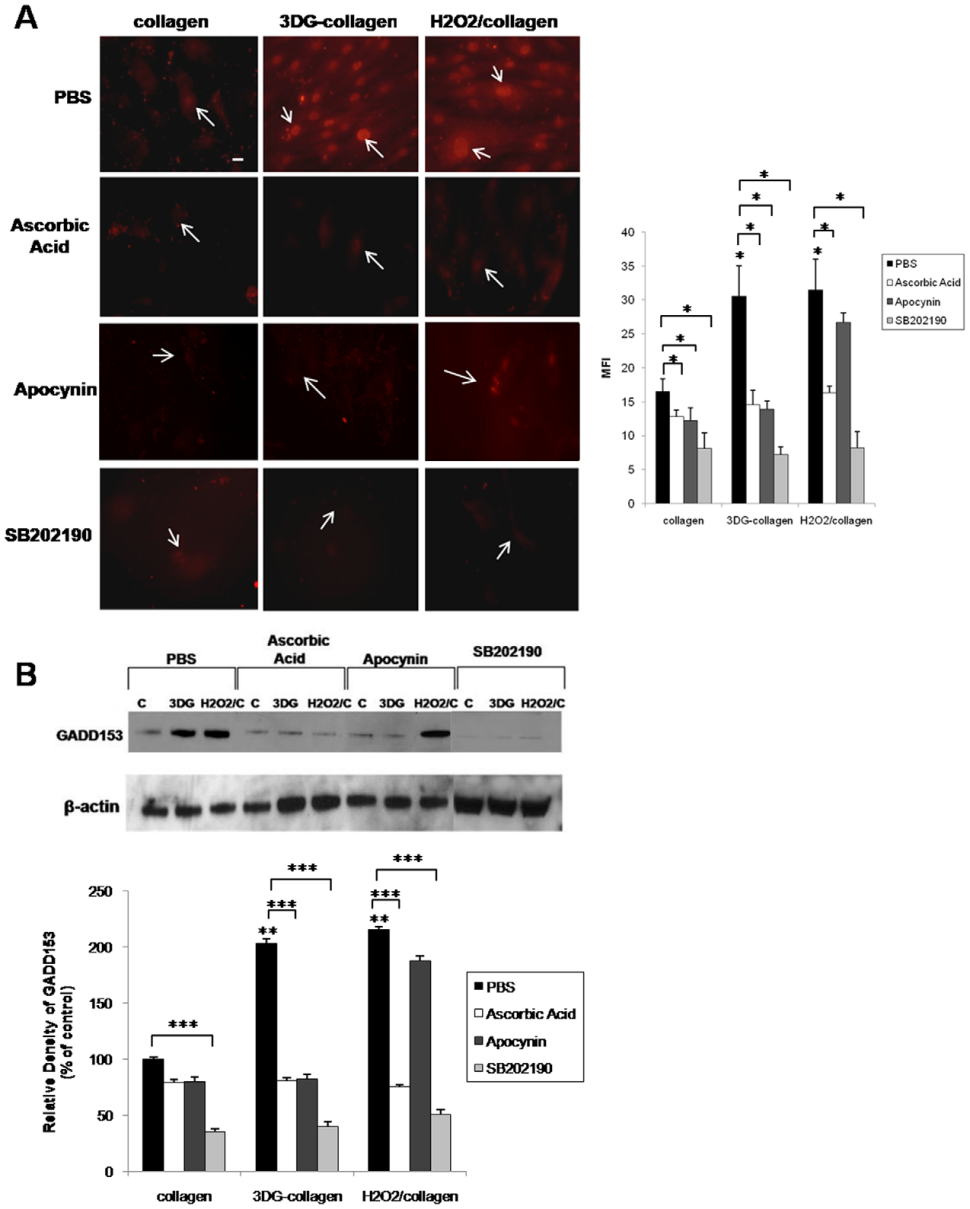
Inhibition of ROS, and p38 MAPK abrogates GADD153 expression in fibroblasts cultured on 3DG-collagen. Fibroblasts were pretreated with 100 µg/mL of ascorbic acid, 1 mM apocynin, or 10 µM SB202190 for 1 h, and cultured on native collagen, 1 mM 3DG-collagen or treated with 50 µM H_2_O_2_ for 24 h. **A**, Fibroblasts were stained and analyzed for the expression of GADD153 in the nucleus by immunofluorescence using a Cy3-conjugated secondary antibody. Representative images were taken at 40× magnification on an epi-fluorescence microscope, and the MFI of ten nuclei was analyzed by Image J. Bars correspond to the MFI of treated fibroblasts. Arrows indicate nuclei containing GADD153. Scale bar represents 10 µm. **B**, Western blot for the expression of GADD153 in whole cell lysates. β-actin was used as a loading control. The bars correspond to the densitometric value of GADD153 after normalization for β-actin. All comparisons are made against collagen treated with PBS unless otherwise indicated. Data are mean±SD (n = 3), ***P<0.0001, **P<0.0005, *P<0.007.

3DG-collagen-induced ROS can lead to phosphorylation of p38 MAPK, which is essential for the activation of GADD153; therefore, the functional role of ROS and p38 MAPK in GADD153 induction was assessed [35], [37], [43], [44]. To determine whether GADD153 induction by 3DG-collagen was a result of free radical-mediated effects, fibroblasts were pretreated with ascorbic acid or apocynin and then cultured on native collagen or 3DG-collagen for 24 h. Fibroblasts cultured on native collagen and treated with H_2_O_2_ were used as a positive control for ROS-induced GADD153 activation. The trafficking of GADD153 from the cytosol to the nucleus was found to be downregulated to 14.6 MFI±2.1 and 16.3 MFI±0.98 in response to ascorbic acid in fibroblasts cultured on 3DG-collagen or native collagen treated with H_2_O_2_, respectively (Figure 6A, p<0.007). The expression of GADD153 in the nucleus of fibroblasts pretreated with apocynin and cultured on 3DG-collagen was also reduced to 13.9 MFI±1.2 (Figure 6A, p<0.007). Western blot was performed to verify the expression of GADD153. GADD153 expression was decreased in response to ascorbic acid and apocynin in fibroblasts cultured on 3DG-collagen, while only ascorbic acid reduced the level of GADD153 in fibroblasts cultured on native collagen treated with H_2_O_2_ (Figure 6B; 81%±2.4% 3DG-collagen treated with ascorbic acid and 82%±4.6% treated with apocynin, and 75%±2.2% H_2_O_2_ treated with ascorbic acid and 188%±3.6% treated with apocynin, p<0.0001). These results suggest that the generation of ROS by Nox4 lies upstream of GADD153.

Next, the role of p38 MAPK in GADD153 activation was assessed in fibroblasts cultured on native collagen or 3DG-collagen. Fibroblasts were pretreated with the p38 MAPK inhibitor SB202190 and cultured on native collagen, 3DG-collagen, or native collagen and treated with H_2_O_2_ for 24 h. Inhibition of p38 MAPK by SB202190 reduced the localization of GADD153 in the nucleus to 7.23MFI±1.13% in fibroblasts cultured on 3DG-collagen (Figure 6A, p<0.007), and reduced the expression of GADD153 to 40%±4.0% (Figure 6A, p<0.0001). In addition, inhibition of p38 MAPK reduced the level of GADD153 expression in fibroblasts grown on native collagen treated with H_2_O_2_ to 51%±3.6% (Figure 6B, p<0.0001), and its nuclear localization to 8.21MFI±2.4 (Figure 6A, p<0.007). These results suggest that the induction of p38 MAPK by upstream ROS is responsible for the activation of GADD153 by 3DG-collagen.

### 3DG-collagen-induced caspase-3 activation is dependent on upstream ROS and p38 MAPK activation

**Figure 7:**
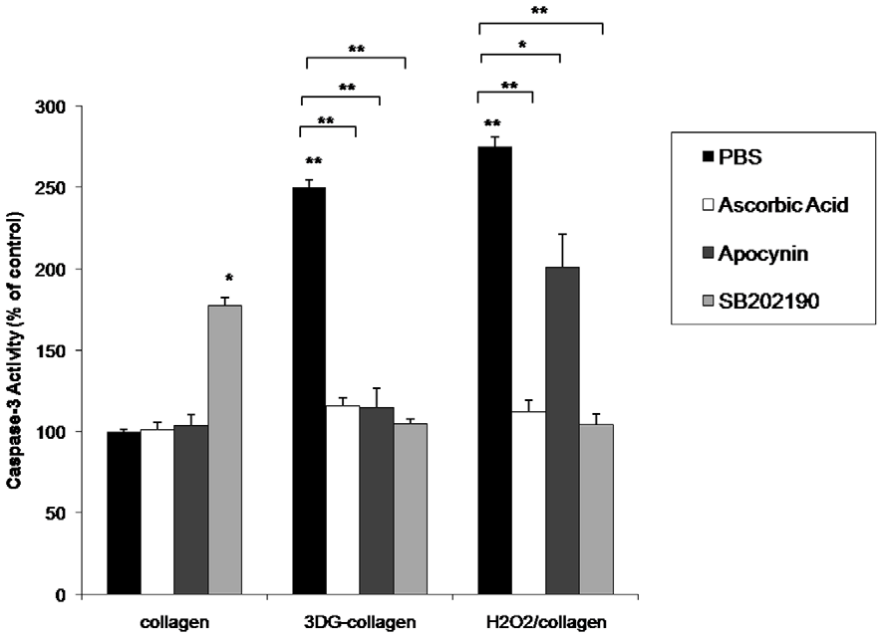
Inhibition of ROS, and p38 MAPK reduces caspase-3 cleavage induced by 3DG-collagen. Fibroblasts were pretreated with 100 µg/mL of ascorbic acid, 1 mM apocynin, or 10 µM SB202190 for 1 h and cultured on native collagen, 1 mM 3 DG-collagen, or treated with 50 µM H_2_O_2_ for 24 h. Treatment of fibroblasts with 50 µM H_2_O_2_ for 24 h was used as a positive control. 100 µg of whole cell lysate was assayed for caspase-3 activity according to the protocol from Caspase-3 Colorimetric Correlate Assay. All samples were performed in triplicate and normalized to the control samples. All comparisons are made against collagen treated with PBS unless otherwise indicated. Data are mean±SD (n = 3), **P<0.0002, *P<0.001.

A caspase-3 assay was performed to determine if ROS and p38 MAPK are responsible for the increased caspase-3 activation observed in fibroblasts cultured on 3DG-collagen. Fibroblasts were pretreated with ascorbic acid, the Nox inhibitor apocynin, or the p38 MAPK inhibitor SB202190; and cultured on native collagen or 3DG-collagen for 24 h, or cultured on native collagen and treated with H_2_O_2_ as a positive control. Fibroblasts treated with ascorbic acid, apocynin, or SB202190 and cultured on 3DG-collagen reduced the activation of caspase-3 to 116%±4.7%, 115%±4.5%, and 105%±2.5% respectively. This expression was comparable to the level of caspase-3 cleavage observed in fibroblasts cultured on native collagen and treated with H_2_O_2_ in the presence of ascorbic acid (112%±7.2%) or SB202190 (104.2%±6.6%), and fibroblasts cultured on native collagen (100%±1.4%; Figure 7, p<0.0002). This data suggests that 3DG-collagen is inducing caspase-3 activation through ER stress, which is dependent on upstream activation of ROS and p38 MAPK through upregulation of Nox4.

### 3DG-collagen induces ROS and apoptosis independent of RAGE signaling

**Figure 8:**
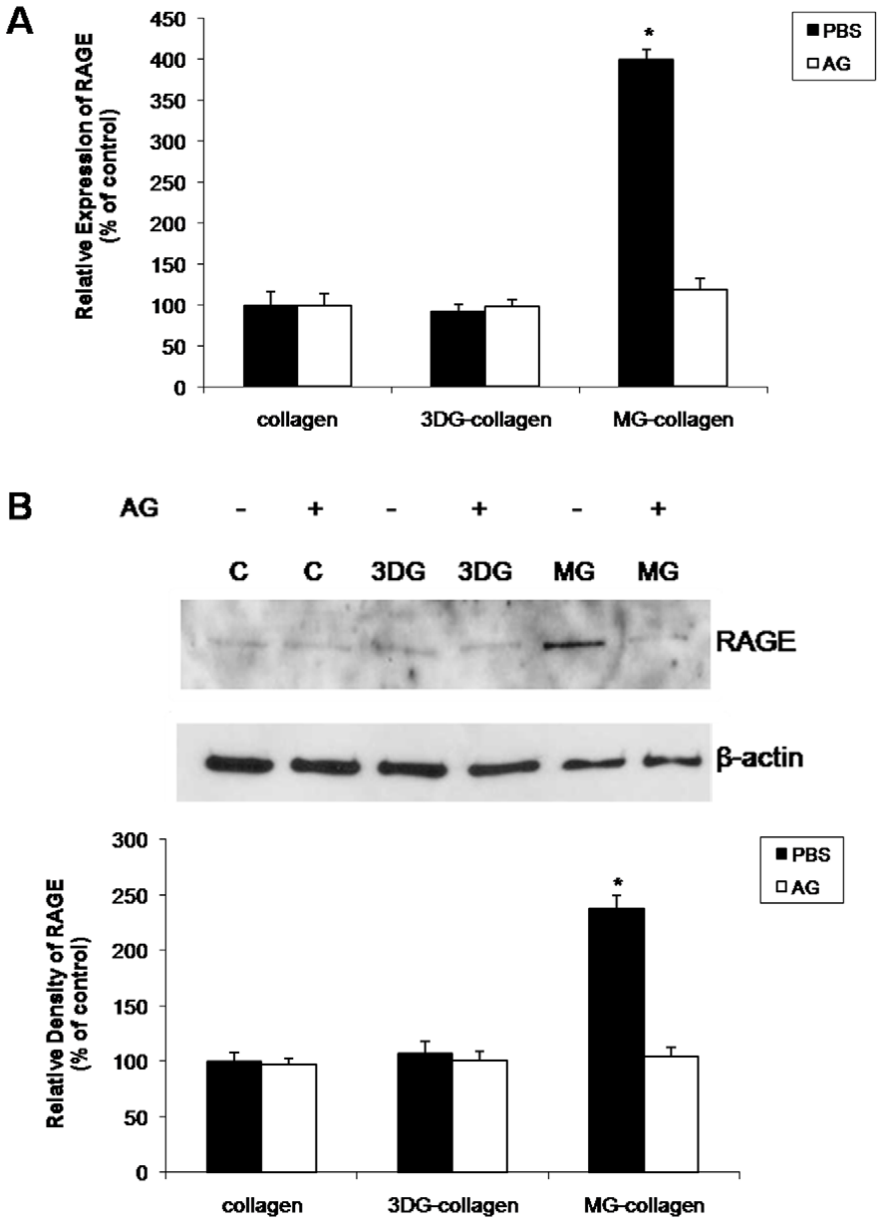
3 DG-collagen does not induce the expression of RAGE. **A**, Fibroblasts were cultured on native collagen, 1 mM 3 DG-collagen, or 1 mM MG-collagen with or without 5 mM AG for 24 h. mRNA was analyzed for the expression of RAGE by real-time PCR. All transcripts were normalized to β-actin. **B**, Fibroblasts were treated as in **A** and analyzed for the expression of RAGE by Western blot. The bars correspond to the densitometric value of RAGE after normalization for β-actin. All comparisons are made against collagen treated with PBS. Data are mean±SD (n = 3), *P<0.002.

When AGEs bind to their receptor, RAGE, ROS are released as a downstream byproduct [25]. To determine if 3DG-collagen is signaling via its interaction with RAGE, we investigated transcript levels of total RAGE. To our surprise, 3DG-collagen did not upregulate RAGE transcript levels (Figure 8A). As a control for the induction of RAGE we cross-linked collagen with MG, which is a well studied AGE precursor known to signal via RAGE [30], [31]. We observed a significant induction of RAGE transcript levels in fibroblasts cultured on MG-collagen; 400%±12% upregulation of RAGE (Figure 8A, p<0.002). Additionally, treatment of MG-collagen with AG reduced the transcript levels of RAGE to 119%±13.3%, which confirms that MG can upregulate RAGE mRNA expression. To ensure that 3DG-collagen is not regulating the RAGE receptor post-transcriptionally we measured RAGE protein levels by Western blot. In contrast to the 238%±11.8% upregulation of RAGE protein in fibroblasts cultured on MG-collagen, 3DG-collagen did not upregulate RAGE protein expression in fibroblasts (107%±10.5%; Figure 8B). To further confirm the specificity of MG, AG abrogated the increase in RAGE protein expression (104%±8.2%) in fibroblasts cultured on MG-collagen (Figure 8B, p<0.002). This data suggests that 3DG-collagen is not upregulating RAGE at both the level of transcription or translation.

**Figure 9:**
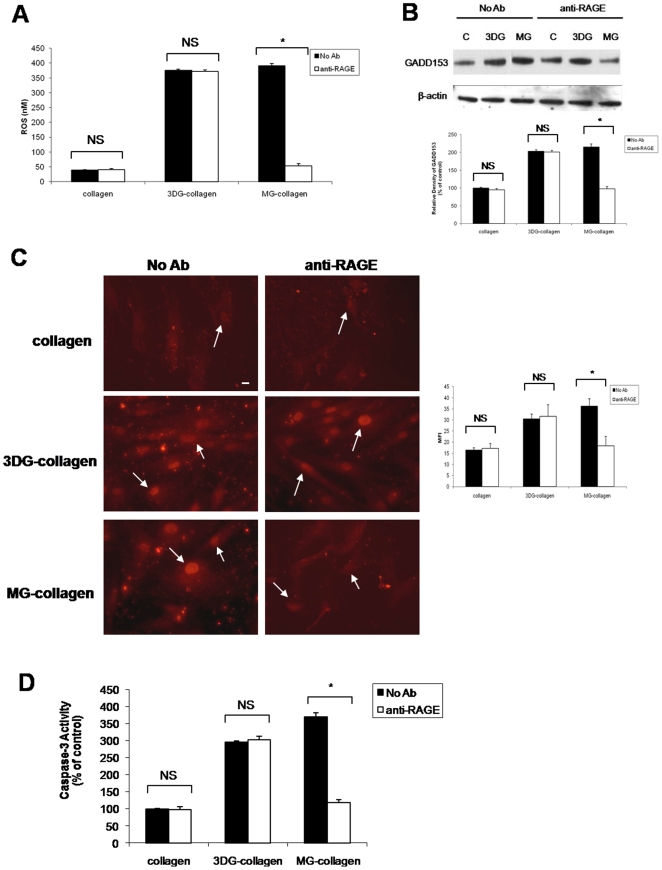
Inhibition of RAGE does not alter the induction of ER stress pathway in fibroblasts cultured on 3 DG-collagen. **A**, Fibroblasts were pretreated with or without the blocking antibody anti-RAGE (10 µg/µL) for 1 h and cultured on native collagen, 1 mM 3 DG-collagen, or 1 mM MG-collagen for 24 h and analyzed for the production of ROS. Fibroblasts were incubated with DCFH-DA for 30 min and the level of intracellular ROS was determined by measuring the fluorescence at 480 nm/530 nm. **B**, Western blot of GADD153 expression after inhibition of RAGE. The bars correspond to the densitometric value of GADD153 after normalization for β-actin. **C**, GADD153 localization in the nucleus was analyzed by immunofluorescence using a Cy3-conjugated secondary antibody. Images were taken at 40× magnification on an epi-fluorescence microscope and the MFI of ten nuclei was processed by ImageJ. Arrows indicate nuclei containing GADD153. Scale bar represents 10 µm. **D**, Caspase-3 activity analyzed according to the protocol from Caspase-3 Colorimetric Correlate Assay. All samples were performed in triplicate and normalized to the control samples. Data are mean±SD (n = 3), *P<0.001.

To further verify the absence of RAGE expression in 3DG-collagen signaling, RAGE was blocked using a blocking antibody specific for the extracellular domain of RAGE, and the levels of ROS were quantified. Intriguingly, the levels of ROS in fibroblasts cultured on 3DG-collagen did not alter after blockade of RAGE. However, we observed the downregulation of ROS with the inhibition of RAGE binding in fibroblasts cultured on MG-collagen (Figure 9A, p<0.001). To further demonstrate that 3DG-collagen signaling was independent of RAGE, we investigated the expression of GADD153. Fibroblasts were pretreated with the RAGE blocking antibody and cultured on native collagen, 3DG-collagen, or MG-collagen; and the level of activated GADD153 was quantified. Blockade of RAGE in fibroblasts cultured on 3DG-collagen did not suppress the activation of GADD153, while GADD153 was suppressed after blockade of RAGE in fibroblasts cultured on MG-collagen (Figure 9B–C, p<0.001). Blockade of RAGE did not decrease the level of caspase-3 activity in fibroblasts cultured on 3DG-collagen, while suppression of caspase-3 activity was observed in fibroblasts pretreated with the RAGE antibody and cultured on MG-collagen (Figure 9D, p<0.001). These results suggest that 3DG-collagen is not signaling through the RAGE receptor as is observed with MG.

### 3DG-collagen activates the ER stress signaling cascade through α1β1 integrin

**Figure 10:**
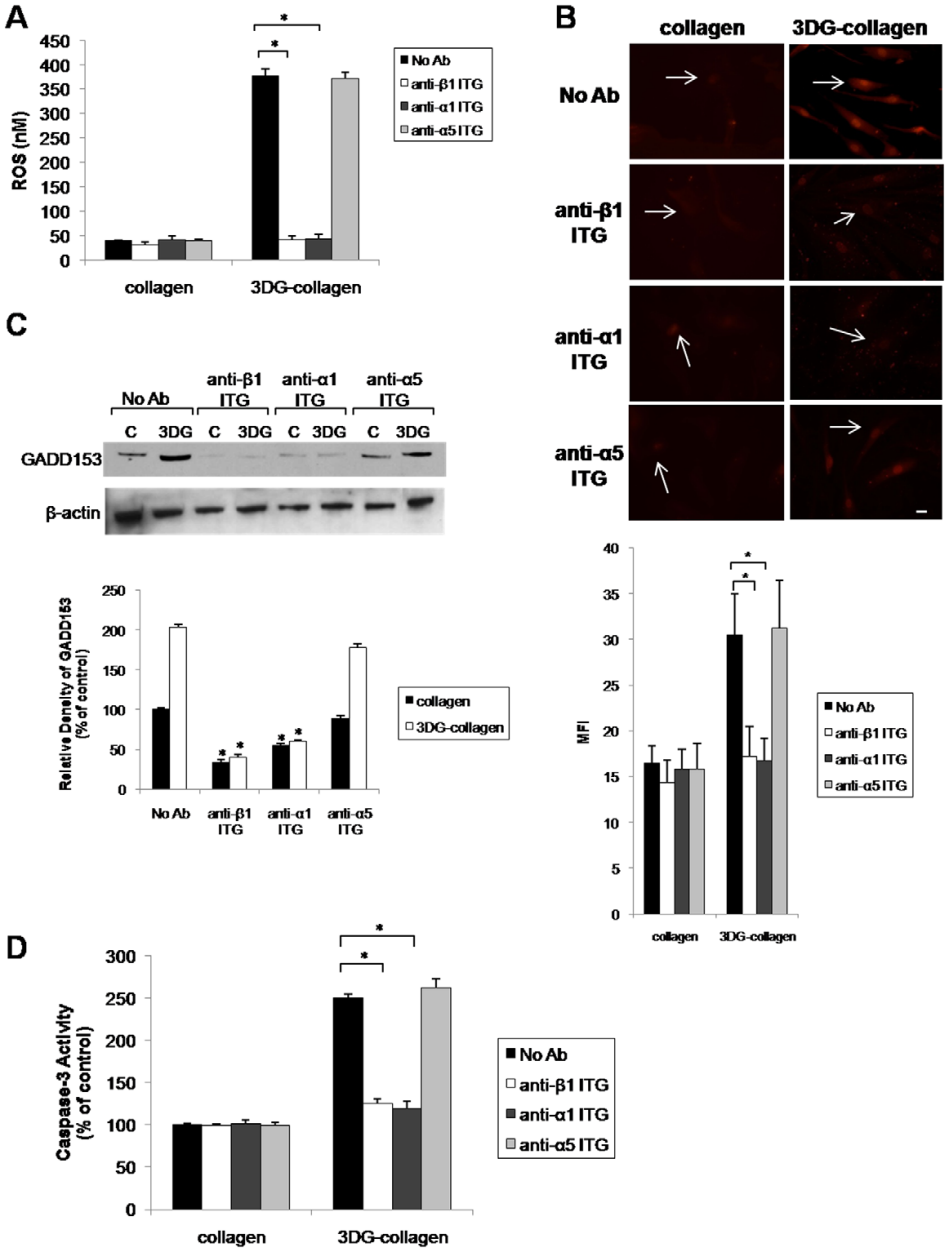
Effect of neutralization of α1β1 integrin on 3 DG-collagen-induced ER stress pathway. **A**, Fibroblasts were pretreated with or without the blocking antibodies anti-β1 ITG, anti-α1 ITG, and anti-α5 ITG (10 µg/µL) for 30 min and cultured on native collagen, or 1 mM 3 DG-collagen, for 24 h and analyzed for the production of ROS. Fibroblasts were incubated with DCFH-DA for 30 min and the level of intracellular ROS was determined by measuring the fluorescence at 480 nm/530 nm. **B**, GADD153 localization in the nucleus was analyzed by immunofluorescence using a Cy3-conjugated secondary antibody. Images were taken at 40× magnification on an epi-fluorescence microscope and the MFI of ten nuclei was processed by ImageJ. Arrows indicate nuclei containing GADD153. Scale bar represents 10 µm. **C**, Western blot of GADD153 expression after neutralization of β1, α1, and α5 integrins. The bars correspond to the densitometric value of GADD153 after normalization for β-actin. **D**, Caspase-3 activity detected using the Caspase-3 Colorimetric Correlate Assay. All comparisons are made against collagen treated with PBS unless otherwise indicated. Data are mean±SD (n = 3), *P<0.001.

To delineate the receptor involved in activating the ER stress pathway by 3DG-collagen, we investigated α1β1 integrin collagen receptor. Previous data has demonstrated that fibroblasts have an increased adherence to 3DG-collagen, which is dependent on binding by α1β1 integrin [28]. Fibroblasts can change their binding affinity for 3DG-collagen, which may cause an overproduction of ROS resulting in increased caspase-3 activation. To verify the role of α1β1 integrin on the ER stress signaling pathway, fibroblasts were pretreated with blocking antibodies against either β1 or α1 integrin and the level of ROS was quantified. α5 integrin, the alpha subunit responsible for binding fibronection, was used as a negative control. Neutralization of both β1 and α1 integrin reduced the production of ROS in fibroblasts cultured on 3DG-collagen to that seen in fibroblasts cultured on native collagen, while neutralization of α5 integrin did not affect the production of ROS (Figure 10A, p<0.001). We next investigated the effect of β1 and α1 integrin neutralization on the expression of GADD153 in fibroblasts cultured on native collagen or 3DG-collagen for 24 h. Blockade of both β1 and α1 integrins suppressed the activation of GADD153 as seen by decreased protein expression and nuclear localization (Figure 10B–C, p<0.001). Moreover, neutralization of β1 and α1 integrin in fibroblasts cultured on 3DG-collagen reduced the activity of caspase-3 to that observed in fibroblasts cultured on native collagen (Figure 10D, p<0.001). These results suggest that the increased binding affinity of α1β1 integrin to 3DG-collagen causes the overproduction of ROS, which in turn leads to increased GADD153 activation and cleavage of caspase-3.

## Discussion

Previous results from our laboratory demonstrated a role for the ER stress pathway in 3DG-collagen signaling; however, the exact signaling mechanism of how 3DG induced ER stress remained elusive [28]. The goal of the present study was to further investigate the induction of caspase-3 activation as an early marker of apoptosis and the signaling events required in fibroblasts cultured on 3DG-collagen. The present study demonstrated that 3DG-collagen induced caspase-3 activation of dermal fibroblasts through activation of GADD153 via induction of ROS and p38 MAPK. Initial studies showed an increase in the apoptotic signaling cascade in fibroblasts cultured on 3DG-collagen, which was confirmed by increases in caspase-3 activity (Figure 1). The level of caspase-3 activity was found to be decreased in fibroblasts cultured on 3DG-collagen and treated with the inhibitors AG and meglumine. Furthermore, we confirmed that 3DG-collagen was inducing caspase-3 activity through induction of ER stress by using an inhibitor of ER stress, salubrinal (Figure 1).

In addition to examining the level of caspase-3 activity, we further investigated the role of ER stress and GADD153 in 3DG-collagen signaling. GADD153 is a marker for misfolded proteins in the ER [37], [43]. It is well documented that ER stress can induce GADD153, which can lead to apoptosis of the cell [33], [34], [37], [43], [46]–[48]. 3DG-collagen induced a significant increase in the level of GADD153, which could be inhibited with AG and meglumine. We confirmed that 3DG-collagen is activating GADD153 through induction of ER stress as salubrinal abolished the 3DG-collagen-induced expression of GADD153 (Figure 2).

To further delineate the signaling mechanism by which 3DG-collagen activates caspase-3, we investigated the expression of ROS. Although ROS are seen as beneficial to the cell during the initial stages of wound healing, chronic activation of ROS have been shown to be stressors on the cell leading to apoptosis [38], [54]. ROS have been shown to induce apoptosis through many pathways, one of which includes ER stress [34], [35], [41]. Additionally, circulating AGEs are known to induce the expression of ROS in many different cell types including neuronal and endothelial cells [15]–[20]. Therefore, we investigated the role of ROS in 3DG-collagen-induced caspase-3 activation in fibroblasts. 3DG-collagen produced ROS, which was attenuated with the addition of the antioxidant ascorbic acid. Inhibitors of 3DG-collagen, AG and meglumine, reduced the level of ROS to that observed in fibroblasts cultured on native collagen suggesting that 3DG is directly responsible for the ROS produced in the fibroblast (Figure 3). To determine the source of increased ROS in fibroblasts cultured on 3DG-collagen we focused our attention to the activation of Nox4. Nox4 has been shown to be highly expressed in fibroblasts [38], [39] and Nox4 has been shown to induce ROS through integrin and growth factor signaling [38]. Fibroblasts cultured on 3DG-collagen produced a significant upregulation in the expression of Nox4 both transcriptionally and translationally (Figure 4A–B). This effect could be inhibited with AG and meglumine suggesting that 3DG-collagen specifically induced Nox4 expression. To determine the role of Nox4 in 3DG-collagen-induced ROS, the Nox inhibitor apocynin was used. Pretreatment of fibroblasts with apocynin reduced the level of intracellular ROS to that observed in fibroblasts cultured on native collagen suggesting that Nox4 is responsible for the production of ROS by 3DG-collagen (Figure 4D).

During times of cellular stress, ROS can induce apoptosis via p38 MAPK activation [35], [37], [43], [44]. p38 MAPK is a stress-activator kinase and has been shown to be an upstream mediator of GADD153 activation [35], [37], [43], [44]. 3DG-collagen-induced phosphorylation of p38 MAPK was found to be dependent on upstream ROS production by Nox4 as the reversal of p38 MAPK phosphorylation occurred when fibroblasts were pretreated with the antioxidant ascorbic acid and the Nox inhibitor apocynin (Figure 5). Moreover, inhibition of ROS and p38 MAPK reduced the level of GADD153 activation and caspase-3 activity of the cell. These data suggest that 3DG-collagen induces an apoptotic signaling cascade through the ER stress pathway, which is dependent on ROS and p38 MAPK activation via Nox4 (Figures 6–7).

It has been well documented that production of intracellular ROS by AGEs induces apoptosis through interaction with its receptor RAGE [19], [23]–[25]. Unlike MG-collagen, a well-studied AGE precursor known to upregulate RAGE, 3DG-collagen did not upregulate the RAGE receptor both transcriptionally or translationally (Figure 8A–B). To provide further evidence that RAGE is not responsible for the ROS-induced caspase-3 activity by 3DG-collagen, a blocking antibody was employed to inhibit the extracellular domain of RAGE. We observed no changes in the levels of ROS, GADD153 expression, or caspase-3 activity when RAGE was blocked suggesting that 3DG-collagen is not signaling through the RAGE receptor (Figure 9A–D). We have previously demonstrated that 3DG-collagen tightly binds to the collagen integrins α1β1, which causes the cell to become static and incapable of migration [28]. Therefore, we proposed that by changing the binding affinity of the fibroblast to collagen, integrins are causing the induction of intracellular ROS, which leads to oxidant stress and caspase-3 activation. We demonstrated that neutralization of the collagen receptors integrin α1 and β1, abrogated the production of ROS by 3DG-collagen (Figure 10A). Additionally, blockade of integrin α1 and β1 reduced the expression of GADD153 and the activation of caspase-3 to that seen in fibroblasts cultured on native collagen (Figure 10 B–D). As a negative control fibroblasts were pretreated with the fibronectin receptor integrin α5, which did not alter the levels of ROS, GADD153 expression, or caspase-3 cleavage (Figure 10). These data suggest that α1β1 integrin on the dermal fibroblast is the receptor responsible for 3DG-collagen-induced caspase-3 activation.

With the growing knowledge of AGEs and their precursors, it is becoming clearer that each AGE and precursor could signal differently depending upon not only the cell type but also the state of the AGE, whether circulating or protein bound. This is believed to be the first documented report to conclusively demonstrate that 3DG-collagen signals independently of RAGE to induce ROS and activate ER stress-induced caspase-3 activation. Because of the number of different AGEs, it is becoming increasingly important to understand how they signal within the cell in order to provide better therapeutics. In addition to the use of AG we studied a new promising therapeutic, meglumine, which decreased caspase-3 activation in the cell by abolishing the production of ROS and activation of GADD153. Additionally, meglumine was shown to reverse the 3DG-collagen mediated effects on fibroblasts by promoting fibroblast migration and proliferation, and increasing ECM production [28], [29]. With the growing number of elderly and diabetic patients, the number of people suffering from diabetic complications associated with AGE formation will continue to increase; therefore, it is vital to gain a better understanding of not only 3DG signaling but of all AGE signaling pathways.

## Methods

This study was approved by the Internal Review Board of Drexel University for human studies.

### Collagen Coating of Cultured Dishes

Acid extracted type I collagen (95–97% COL1A1; 3–5% COL3A1) from human skin was purchased from Stem Cell Technologies (Vancouver BC, Canada). The collagen was diluted in PBS to a final concentration of 0.067 mg/ml. The diluted collagen was added to the tissue culture dish for 2 h at 37°C as previously described [28]. The culture dish was washed 3 times with 5 mL of sterile PBS to remove any nonadherent collagen from the dish. To modify the matrices, collagen coated dishes were incubated overnight with 1 mM 3 DG or 1 mM MG, and/or 5 mM AG, which was added simultaneously to the collagen. Unincorporated 3 DG, MG, or AG was removed by gently washing the collagen three times with sterile PBS prior to plating with fibroblasts. For treatment with H_2_O_2_, fibroblasts were cultured on native collagen followed by the addition of 50 µM H_2_O_2_ for 24 h.

### Tissue Culture

Normal human dermal fibroblasts from individuals (GM06120, GM00498, GM04190) aged 3–85years old (less than passage 15) were purchased from the Coriel Institute (Camden, NJ). Unless otherwise noted, fibroblasts were seeded onto native collagen and 3DG-collagen coated dishes and cultured until 70% confluent in Dulbecco's Modified Eagle's Medium (DMEM) supplemented with 10% dialyzed FBS and 1% penicillin/streptomycin.

### Chemicals and Antibodies

Salubrinal was purchased from Calbiochem (La Jolla, CA). SB202190 and ascorbic acid were purchased from Sigma (St. Louis, MO). 6-Carboxy-2′,7′-dichlorofluorescin diacetate (DCFH-DA) was purchased from Cell Biolabs Inc. (San Diego, CA). H_2_O_2_ was purchased from Fisher Scientific (Pittsburgh, PA). Apocynin, the monoclonal antibody against GADD153, and polyclonal antibodies against Nox4 and RAGE were purchased from Santa Cruz Biotechnology (Santa Cruz, CA). Polyclonal antibodies against phospho-p38 MAPK, total p38 MAPK, and β-actin were purchased from Cell Signaling Technologies (Danvers, MA). Secondary antibodies were purchased from Jackson Labs (West Grove, PA). Meglumine-HCl was a kind gift from Dynamis Therapeutics, Inc (Jenkintown, PA).

### Inhibition of ROS, p38 MAPK, GADD153, and RAGE

For inhibition studies, fibroblasts were cultured until 70% confluent, trypsinized, preincubated for 1 h with or without the antioxidant ascorbic acid (100 µg/mL), the ER stress inhibitor salubrinal (40 µM), the p38 MAPK inhibitor SB202190 (10 µM), the Nox4 inhibitor apocynin (1 mM), or the blocking antibody against RAGE (10 µg/µL), and replated onto collagen, 3DG-collagen, and MG-collagen coated dishes or treated with 50 µM H_2_O_2_ for 24 h in DMEM containing 1% FBS and 1% penicillin/streptomycin. The concentrations of inhibitors are similar to doses used in previously published studies [8], [21], [22], [55]–[58].

### Integrin Neutralization

Integrin neutralization assays were performed using blocking antibodies against integrins β1, α1, and α5 (Santa Cruz, CA) according to [59] to determine the involvement of integrins in ROS production, GADD153 activation, and caspase-3 cleavage. 70% confluent cells were suspended in 1% FBS-DMEM, incubated with antibodies (10 µg/µL) for 30 min at 37°C and then plated on chamber slides coated with collagen or 1 mM 3DG-collagen for 24 h.

### SYBR Green Quantitative RT-PCR

Cells were harvested and RNA was extracted using the RNeasy Mini kit (Qiagen, Valencia, CA) according to manufacturer's protocol. To verify expression of Nox4 and RAGE; 2.0 µg of total RNA was reverse-transcribed using Superscript-III reverse transcriptase (Invitrogen Carlsbad, CA), according to manufacturer's protocol. Transcripts were quantified using SYBR green PCR amplification (Qiagen). All mRNA transcripts were normalized to β-actin expression. The following primers were employed to detect transcripts of interest:

Nox1-forward: 5′-TTCACCAATTCCCAGGATTGAAGTGGATGGTC-3′

Nox1-reverse: 5′-GACCTGTCACGATGTCAGTGGCCTTGTCAA-3′;

Nox2-forward: 5′-AACGAGCAGGCGCTGGCGTCC-3′

Nox2-reverse: 5′-GCTTGGGCTCGATGGGCGTCCACT-3′;

Nox4-forward: 5′-CTGGAGGAGCTGGCTCGCCAACGAAG-3′

Nox4-reverse: 5′-GTGATCATGAGGAATAGCACCACCACCATGCAG-3′;

RAGE-forward: 5′-CAGGACCCTGGAAGGAAGCA-3′

RAGE-reverse: 5′-TGATGGATGGGATCTGTCTGTG-3′;

β-actin-forward 5′-TTGCCGACAGGATGCAGAA-3′

β-actin-reverse 5′-GCCGATCCACACGGAGTACTT-3′.

### Immunofluorescence

Cells cultured in chamber slides for 24 h at 30% confluency were fixed in 4% paraformaldehyde for 10 min. Cells were incubated in a 1∶50 dilution of GADD153 or Nox4 antibodies and incubated in a humid chamber at room temperature for 60 min. The cells were washed 3 times with PBS and then stained with Cy3 or Cy2 secondary Ab (1∶50 dilution) (Jackson Labs) in a humid chamber at room temperature for 40 min. Cells were washed 3 times with PBS and mounted with DAPI. Images were viewed with an epi-fluorescence microscope at 40× magnification. Ten images from each preparation were taken. For GADD153 analysis, the mean fluorescence intensity (MFI) of the nuclei of each cell was calculated using ImageJ.

### Western blot

Cells were harvested and protein was extracted using 100 µL of cell lysis buffer supplemented with 0.3% PMSF and proteinase and phosphatase inhibitors. 100 µg of protein from each sample was size fractionated on 10% SDS PAGE gels (Invitrogen) for 60 min at 180 volts. The proteins were transferred to PVDF membrane and the membrane blocked with 5% skim milk. The PVDF was probed with an antibody directed against either GADD153 (1∶200), Nox4 (1∶200), RAGE (1∶500), phospho-p38 MAPK (1∶1000), total p38 MAPK (1∶1000), or β-actin (1∶1000). The membrane was washed with TBS-Tween to remove any unbound proteins and incubated with the secondary antibody, goat-anti-rabbit-HRP (1∶2000). The signal was developed with SuperSignal Chemiluminescent Substrate (Pierce, Rockford, IL).

### Reactive Oxygen Species Assay

Cells were cultured in a 96-well collagen or 3DG-collagen-coated plates for 24 h. As a positive control, cells were cultured on native collagen and treated with 50 µM H_2_O_2_ for 24 h. The level of ROS was then quantified according to the manufacturer's protocol (Cell Biolabs, Inc., San Diego, CA). The cells were treated with 1× DCFH-DA solution in DMEM for 30 min at 37°C, washed with PBS, and the assay was terminated by the addition of 2× Cell Lysis Buffer. 150 µL of the lysis mixture was added to a 96-well plate and the fluorescence of the lysate was measured at 480 nm/530 nm on a Fluoroskan Ascent FL (LabSystems, Beverly, MA).

### Caspase-3 Assay

Cells were harvested and lysed in cell lysis buffer as described above. Whole cell lysates were combined with Caspase-3 substrate reaction buffer and incubated for 3 h at 37°C and the absorbance was measured at 450 nm with a plate reader according to the manufacturer (Assay Designs, Ann Arbor, MI). Background readings from cell buffers and substrates were subtracted from the sample readings. A standard curve was used to calculate the increase in caspase-3 activity.

### Statistical Analysis

The results are mean±SD. The resulting data were subjected to either a two-tailed paired *t*- test for comparison between two groups, or a one-way ANOVA for comparison between multiple groups followed by Tukey's post-hoc test. A P value<0.05 was considered significant.
